# Signalling with a cryptic trait: the regularity of barred plumage in common waxbills

**DOI:** 10.1098/rsos.160195

**Published:** 2016-05-04

**Authors:** Cristiana I. J. Marques, Helena R. Batalha, Gonçalo C. Cardoso

**Affiliations:** CIBIO—Centro de Investigação em Biodiversidade e Recursos Genéticos, Universidade do Porto, Campus Agrário de Vairão, 4485-661 Vairão, Portugal

**Keywords:** camouflage, communication, ornamentation, pigmentation pattern, sensory ecology, sexual selection

## Abstract

Sexual signals often compromise camouflage because of their conspicuousness. Pigmentation patterns, on the contrary, aid in camouflage. It was hypothesized that a particular type of pattern—barred plumage in birds, whereby pigmented bars extend across feathers—could simultaneously signal individual quality, because disruptions of these patterns should be perceptually salient at close range and help assess plumage condition. Here we show that common waxbills (*Estrilda astrild*), which have extensive barred plumage, have more regular patterns as adults than as juveniles, and that adult males have more regular patterns than females. Both these differences are indicative of sexual signalling in species with conventional sex roles. More regular barred plumage was related to better body condition in adult males. Colour ornamentation traits were also related to aspects of quality, either the same as barred plumage (body condition) or a different one (good feather development), supporting both the ‘redundant message’ and the ‘multiple message’ hypotheses for the coexistence of multiple sexual signals. Although receiver responses to the regularity of barred plumage were not studied here, research on other species has shown that barred plumage can mediate social interactions. We conclude that using barred plumage as a signal of quality helps circumvent the functional compromise between camouflage and communication.

## 1. Background

Many sexual signals of animals, such as vivid colours, strong scents or loud songs, are conspicuous and can attract the attention of predators [1]. Camouflage, on the contrary, consists of traits that decrease the detectability of animals. Many animals manage a compromise between the two by modulating signalling: for example, hiding colour patches when not signalling actively, or withdrawing from signalling when predation risk is higher [2]. An intriguing alternative possibility is that some camouflage traits also accommodate signalling components, thus overcoming the functional compromise between camouflage and signalling. For example, blue and yellow patterns common in reef fishes are conspicuous at close range but blend into a cryptic colour at a distance [3].

It has been suggested that a particular type of pigmentation pattern in birds, barred plumage, can accommodate both camouflage and signalling components [4–6]. Barred plumage consists of alternating darker and lighter bars transversally across each feather, and is common among bird species [7]. Pigmentation patterns often serve as camouflage or motion camouflage, as they are more difficult to perceive or track against heterogeneous backgrounds (e.g. [8,9]; reviewed in table S1 of [10]). Barred plumage is a special type of pattern, because the precise arrangement of barred feathers, side by side, forms a more or less continuous visual pattern, on which irregularities (e.g. owing to broken or worn feathers, or to differences in development among feathers) would be perceptually salient at close range. Therefore, in the context of sexual selection, barred plumage might function as an amplifier signal of plumage quality and, thus, individual quality.

Indirect evidence suggests that barred plumage is used for both camouflage and communication. Across avian species, barred plumage is less frequent in adult males than in females or juveniles [6], which is consistent with camouflage being its most common function because, in species with conventional sex roles, adult males often express less camouflaged coloration in favour of ornamental coloration. However, there are also species where only males or only adults express pigmentation patterns, and this is more frequent for barred plumage than for mottled plumage, which lacks a precise and regular arrangement of pigmentation, indicating that barred plumage can also evolve or be maintained owing to sexual signalling [6]. As noted above, a putative explanation for barred plumage to function as a sexual signal is its regular arrangement of bars, which could advertise plumage and individual quality. As yet, differences in the regularity of barred plumage among individuals and its information content have never been studied.

Here, we studied the regularity of barred plumage in wild common waxbills (*Estrilda astrild*). The common waxbill is a gregarious and mutually ornamented finch, with vivid red colour in the bill, mask and breast, and barred plumage covering most of the body; juveniles do not have red bills, but have barred plumage and red colour in the mask and breast [11]. As is typical of ornamentation in species with conventional sex roles, adult males have a larger extent and saturation of red plumage than females, although the degree of male and female ornamentation overlap extensively [12], and juveniles have on average a lower extent and saturation of red plumage [13].

We quantified the regularity of barred plumage as the degree of deviation in relation to perfectly delineated and continuous bars [5], and used these data to test three predictions of the hypothesis that regular barred plumage functions as a sexual signal of quality. If regular barred plumage evolved in part owing to sexual signalling, then we predict (i) that adults should have more regular patterns than juveniles and (ii) that adult males should have more regular patterns than adult females, as happens with the greater extent and saturation of their red colour ornamentation. We also tested if (iii) the regularity of barred plumage in adults indicates aspects of individual quality, such as body condition, fault bars in feathers or ectoparasitic load. In addition, on the assumption that barred plumage functions as a sexual signal, we also asked if its coexistence with red colour ornamentation is best explained by the ‘multiple message’ or the ‘redundant message’ hypotheses for multiple ornaments [14]. For this, we tested if the regularity of barred plumage correlates with red ornamentation, and if red ornamentation indicates the same or different aspects of individual quality compared with barred plumage.

## 2. Material and methods

### 2.1. Subjects, colour ornamentation and individual quality

We studied common waxbills in 23 sites across the range of their biological invasion of Portugal, having captured over 600 waxbills. We could sex molecularly, age and take good quality photographs of barred plumage (see below) from 478 of these, which form the dataset used here. We refer to Carvalho *et al.* [15] for a description of sites, field methods, age assessment and molecular sexing, and to Cardoso *et al.* [13] for a description of colour measurements. Of the colour measurements made, here we use those that show sex or age differences: saturation of red colour in the bill and breast (quantified as the proportion of reflectance in the range where carotenoid pigments reflect the most, relative to the bird visible range; reflectance from 500 to 700 nm wavelengths/reflectance from 320 to 700 nm wavelengths), and extent of red colour in the mask and breast (quantified as area of red coloration in the breast, or area of red in the mask standardized by head size); brightness of red colour did not differ between the sexes or age classes [13] and is not used here.

To compute an index of body condition, we weighed each bird (with a digital or a spring balance to the nearest 0.1 g) and took three measurements of skeletal size: tarsus length (from the notch at the intertarsal joint to the last scale before toes diverge), bill height (at the point of feathering) and total head length (from tip of bill to furthest point at the back of head; all measured with callipers to the nearest 0.1 mm). We computed an index of body condition as the residual mass from a linear regression of body mass on body size (*R*^2^ is 0.23), having used as a measure of body size the first principal component (PC) from a principal component analysis (PCA) on the three skeletal measurements (50.2% variation explained, trait loadings for tarsus is 0.62, for bill depth is 0.71 and for head length is 0.79).

We assessed the load of feather mites by summing the number of tail and wing feathers with mites, each multiplied by a code of the extent of infestation, adapted from Behnke *et al.* [16,17]: 0, no mites; 1, only close to the shaft, in less than half its extension; 2, along the shaft in more than half its extension; 3, more than half feather's extension and often extending away from the shaft towards the edges. We similarly assessed the extent of fault bars by summing the number of tail and wing feathers with fault bars, each multiplied by a code of its extent: 0, none; 1, only incomplete fault bars; 2, one complete fault bar (i.e. across the width of the feather); 3, more than one complete fault bar. Owing to occasional logistic issues, data on aspects of quality are missing for some individuals; sample sizes using these data are indicated for each analysis.

### 2.2. Regularity of barred plumage

**Figure 1.**
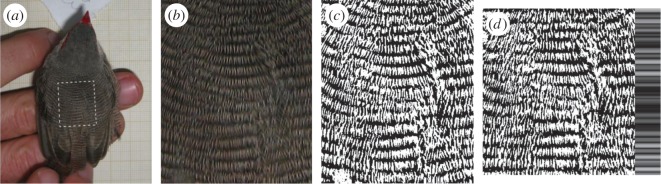
Example of (*a*) a dorsal photograph of common waxbill plumage, (*b*) a sample of plumage for analysis (marked in panel (*a*) with a dashed rectangle), which is then (*c*) converted to dichromatic and (*d*) corrected for perspective and curvature. The shades of grey on the right side of panel (*d*) code for colour homogeneity within each line of pixels (bright for high homogeneity and dark for low homogeneity); the regularity of barred plumage is assessed as the average homogeneity for each line of pixels [5].

Common waxbills have barred plumage both dorsally and ventrally. Quantification of the regularity of barred plumage is best done using large areas of plumage [5]. Therefore, because ventral barred plumage is interrupted by the ornamental red coloration and can be disarranged in the flanks by wing movements, we chose to quantify the regularity of dorsal barred plumage. After removing each bird from the capture bag and banding, and prior to morphological measurements and others, we took photographs in standardized conditions. We used a 10 MP Canon iXus 85 IS camera, mounted 23 cm high on a tripod pointing downward, on a table in the shade, with no zoom and using the camera's onboard flash. Each bird was held by the legs on the table such that its dorsal surface was approximately horizontal, and its gaze directed with a hand gesture, so that it faces approximately forward (figure 1*a*).

On image-processing software, we rotated these dorsal photographs so that the bars were approximately horizontal, and cropped a rectangle of mantle plumage. To standardize the size and shape of these rectangular areas of plumage, in 20 randomly chosen birds, we selected a rectangle from the base of the nape (the nape has distinctively narrower bars) towards the end of the mantle (before the wing flight feathers cover the mantle), and of width identical to that separating the two ends of the red masks. Selecting an area of plumage based on such landmarks is influenced by slight differences in posture between birds. Therefore, we used the average dimension of these 20 rectangles as the standard size for analysis of regularity, and for all birds, we selected a rectangle with these dimensions centred relative to the mantle. We excluded images with photographic problems (blurred or overexposed) or when, owing to the posture of the bird, the flight feathers, the cheek white patch or the distinctive pattern of the nape were included in the rectangular sample, resulting in a total of 478 waxbills (231 and 193 adult males and females, and 27 and 27 juvenile males and females).

**Figure 2.**
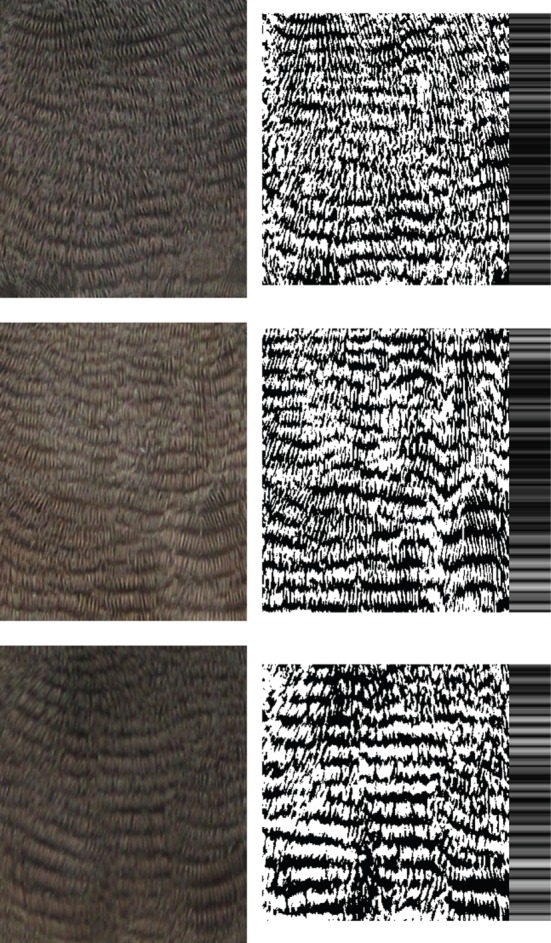
Examples of plumage patterns differing in regularity. The examples are from adult males, have an identical proportion of black pixels and, from top to bottom panels, were scored in the lowest third, middle third and highest third of percentiles of REG. See the main text and figure 1*b* and *d* for an explanation of image processing.

These rectangular samples of barred plumage were processed using an algorithm [5] that first converts colour images to black-and-white while correcting for differences in shading across the surface, then corrects for curvature and perspective of the image, and finally computes a measure of regularity (hereafter REG) by comparison with a pattern of perfectly delineated and continuous bars (figure 1). REG may vary from 1, when every row of pixels is completely monochromatic (i.e. all bars are unbroken, with perfectly smooth and parallel contours; note that perfect regularity is biologically unattainable) to 0, when every row of pixels has an equal amount of dark and light pigmentation (the minimum value of REG attainable also depends on the relative amounts of dark and light pigmentation in the pattern). Examples of plumage patterns differing in regularity are given in figure 2. The algorithm also provides an efficiency metric (EM) for the conversion to black-and-white, as the proportion of brightness variation in the original image that was retained in the conversion. High EM indicates that the original image was very dichromatic (as expected for barred plumage, which alternates one dark and one light colour), and low EM may indicate image problems such as strongly heterogeneous shading; it is therefore advisable to verify if differences in this EM influence the calculation of REG [5]. Additionally, in this type of very fine barred plumage, clear bars are interrupted by the small shadows in between barbules, whereas dark bars are not interrupted by these shadows (figure 1), and therefore small differences among individuals in the width of melanized bars might affect the computation of REG. To address this, we computed the proportion of black pixels in the aligned dichromatic images of barred plumage (figure 1*d*), and assessed if these differences in melanization affect the computation of REG.

In our sample, EM varied between 0.62 and 0.74, there were no outliers, and EM was not correlated to REG (*r* = 0.07, *n* = 478, *p* = 0.12). The proportion of black pixels varied between 0.47 and 0.68 and was correlated to REG (*r* = 0.39, *n* = 478, *p* < 0.001), particularly in juveniles (males: *r* = 0.55, *n* = 27, *p* = 0.003; females: *r* = 0.55, *n* = 27, *p* = 0.003), but also in adults (males: *r* = 0.29, *n* = 231, *p* < 0.001; females: *r* = 0.45, *n* = 193, *p* < 0.001). Therefore, the metric REG is influenced by both the regularity of the barred patterns plus differences in the degree of melanization of these patterns and, rather than analysing the raw values of REG, we analysed residual REG (hereafter resREG) obtained from linear regression of REG on the proportion of black pixels. This is a metric of the regularity of the barred pattern after removing the effect attributable to differences in melanization. For analyses comparing age classes, we used residuals from a regression with all 478 birds; for analyses comparing the adult sexes, we used residuals from a regression with all adults; and for analyses run on each adult sex separately, we used residuals from regressions with adult males or females only.

### 2.3. Analyses of residual regularity

Sex ratios in our sample were similar for adults and juveniles, and the distribution of resREG closely approximated normality. Therefore, we tested for age differences in resREG with a simple *t*-test. We compared resREG between adult males and adult females with a *t*-test.

We tested whether resREG was related to measures of condition separately for adult males and females. We used general-linear models (GLM) with resREG as the dependent variable and, as predictors, the three measures of condition (body condition, ectoparasite load and intensity of fault bars) and two additional geographical variables. These two geographical variables are the first and second PCs from a PCA on ecological differences among sites (hereafter geography PC1 and geography PC2), taken from previous work on this system, which reflect biologically relevant ecological gradients: high PC1 scores indicate low altitude, warm and dry sites with higher densities of waxbills, and high PC2 scores indicate sites with strong climatic seasonality (see [15] for details). Because ornamentation and behaviour change with these ecological gradients [13,15], we included the geography PCs in statistical models in order to control for similar ecological effects when testing for relations between barred plumage and the quality of individuals. Eventual ecological effects detected in these tests should be interpreted cautiously, because ecology varies among sites, rather than among individuals, and our individual-level analyses are therefore permissive.

Because we have multiple predictors in these models, we performed model selection based on Akaike's information criterion (AIC). We calculated AIC corrected for small sample sizes (AICc) for every combination of predictor variables, and selected the models that are statistically indistinguishable from the best (hereafter, the ‘best models’; i.e. those within two AICc units from the model with lowest AICc [18,19]). In cases where the null model (the model with no predictors) is within two AICc units from the best model, then there is no evidence for any predictor having explanatory power and we did not select best models. We computed the relative importance (RI) of each predictor by summing the Akaike weights of the best models in which a given predictor is included [19]: predictors included in all best models have a RI of 1, and predictors that are rarely included have low RI. In order to translate these results into a more conventional statistical approach, we also calculated a model-averaged standardized partial regression coefficient (${\hat{\bar{\beta }}}_{\mathrm{st}}$) for each predictor included in the best models. This is done by averaging the standardized partial coefficients across all best models, weighted by the Akaike weight of each model [19], and then using it for conventional hypothesis testing [18].

### 2.4. Analyses of colour ornamentation

We asked if resREG is related to colour ornamentation, and if the potential information conveyed by resREG is redundant or distinct from the information conveyed by colour ornamentation.

We tested whether resREG is related to colour ornamentation separately for adult males and females, using GLM with resREG as dependent variable and, as predictor variables, the four measures of ornamental coloration: the extent of red in the breast and mask, and red colour saturation in the breast and bill. The extent of red coloration was measured in birds from all field sites, and colour saturation only for birds in 11 of the sites (see [13]). Therefore, the sample size for this analysis is limited by the availability of colour saturation data. Because we have multiple predictors that may be related to some extent, AICc-based model selection was performed as described above, which selects the best predictors of resREG to the exclusion of redundant predictors.

We tested whether colour ornamentation was related to measures of condition with GLM similar to the ones described in §2.3, with the difference that the dependent variable was one of the measures of colour ornamentation (mask area, red area in the breast, red saturation in the bill or breast). As before, the predictor variables were the measures of condition (body condition, ectoparasite load and fault bars intensity) along with geographical variables PC1 and PC2. Model selection and model averaging were done as described before. Statistical analyses were done on IBM SPSS Statistics 21, except for AIC-based model selection, averaging and testing model-averaged coefficients, which used the ‘MuMIn’ package [20] in R v. 3.1 [21].

## 3. Results

### 3.1. resREG

**Figure 3.**
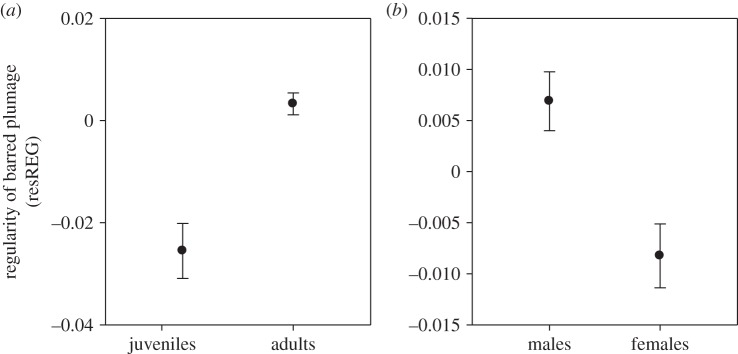
Mean regularity of barred plumage (resREG ± s.e.) for (*a*) juvenile versus adult waxbills, and (*b*) male versus female adult waxbills.

As predicted by the hypothesis that the regularity of barred plumage evolved as a sexual signal, adults had on average higher resREG scores than juveniles (*t*_476_ = 4.559; *p* < 0.001; figure 3*a*) and, among adults, males had higher resREG than females (*t*_422_ = 3.561; *p* < 0.001; figure 3*b*).

For adult males, AICc-based model selection found two statistically indistinguishable best models for predicting resREG (electronic supplementary material, table S1), and the null model was not among the best models (ΔAICc = 6.38). Both best models included positive effects of body condition and of geography PC2 (RI for both predictors = 1); a negative effect of fault bars intensity was also present in one of the best models (RI = 0.28; table 1 and electronic supplementary material, table S1). Effect sizes of predictors, calculated using an averaged model, indicate that body condition and PC2 were significantly related to resREG (${\hat{\bar{\beta }}}_{\mathrm{st}}=0.15,\;$ *p* = 0.03 and ${\hat{\bar{\beta }}}_{\mathrm{st}}=0.18,\;$ *p* = 0.007, respectively; table 1).

**Table 1.** Relative importance (RI) and partial regression coefficients (${\hat{\bar{\beta }}}_{\mathrm{st}}$) from model-averaging of GLMs relating measures of condition and geographical–ecological gradients to the regularity of barred plumage (resREG) in adult waxbills.

		male resREG (*n* = 218)	female resREG (*n* = 184)
body condition	RI	1	0.82
	${\hat{\bar{\beta }}}_{\mathrm{st}}$	0.15 (*p* = 0.03)	0.13 (0.07)
ectoparasites	RI	—	0.34
	${\hat{\bar{\beta }}}_{\mathrm{st}}$		−0.09 (0.24)
fault bars	RI	0.28	0.20
	${\hat{\bar{\beta }}}_{\mathrm{st}}$	−0.03 (0.69)	−0.07 (0.36)
geography PC1	RI	—	0.66
	${\hat{\bar{\beta }}}_{\mathrm{st}}$		−0.12 (0.11)
geography PC2	RI	1	—
	${\hat{\bar{\beta }}}_{\mathrm{st}}$	0.18 (0.007)	

For adult females, model selection found eight best models (electronic supplementary material, table S2), and the null model was not among these (ΔAICc = 2.05). The strongest effect was that of body condition, present in six of the best models (RI = 0.82), followed by effects of geography PC1 (present in five models, RI = 0.66), ectoparasites and the extent of fault bars (present in three and two models, respectively, both RI < 0.5; table 1 and electronic supplementary material, table S2). Using an averaged model, only body condition had a marginal positive effect on resREG (${\hat{\bar{\beta }}}_{\mathrm{st}}=0.13,\;$ *p* = 0.07; table 1).

### 3.2. Colour ornamentation

resREG was not significantly related to ornamental colour traits because, both for adult males and females, the null model was included within the set of statistically indistinguishable best models (i.e. within two AICc from the best model; ΔAICc = 1.92 in males, and 0.32 in females).

For some colour ornamentation traits (extent and saturation of red colour in the breast of adult males or females, and saturation of red in the bill of adult females), we found no significant relations with predictors; in all cases, the null model was part of the set of statistically indistinguishable best models (ΔAICc = 0.99, 1.81, 0, 0.92 and 0, respectively). The electronic supplementary material, tables S3–S5 show the best models for the relations of the remaining three colour traits and aspects of individual condition. Overall, there were positive effects of body condition, ectoparasite load and geography PC1 on male red bill saturation, all with RI of 1 and significant coefficients estimated from the averaged model (table 2). The area of the red mask in males was negatively related to the extent of fault bars (RI = 1 and significant coefficient from the averaged model, table 2). The area of the red mask in females, on the contrary, was positively related to the extent of fault bars (RI = 0.6), though the coefficient from the averaged model was not significant (table 2), and was significantly influenced by geography PC1 and PC2 (in both cases RI = 1, table 2). The remaining predictors in the best models for these colour traits had RI lower than 0.7, and non-significant coefficients estimated from averaged models (table 2).

**Table 2.** Relative importance (RI) and partial regression coefficients (${\hat{\bar{\beta }}}_{\mathrm{st}}$) from model-averaging of GLMs relating measures of condition and geographical–ecological gradients to colour ornamentation in adult waxbills.

		male red bill saturation (*n* = 92)	male red mask area (*n* = 218)	female red mask area (*n* = 184)
body condition	RI	1	—	0.41
	${\hat{\bar{\beta }}}_{\mathrm{st}}$	0.21 (*p* = 0.03)		0.08 (0.25)
ectoparasites	RI	1	0.20	—
	${\hat{\bar{\beta }}}_{\mathrm{st}}$	0.28 (0.005)	0.04 (0.60)	
fault bars	RI	0.3	1	0.61
	${\hat{\bar{\beta }}}_{\mathrm{st}}$	−0.08 (0.46)	−0.18 (0.01)	0.12 (0.09)
geography PC1	RI	1	0.52	1
	${\hat{\bar{\beta }}}_{\mathrm{st}}$	0.21 (0.04)	0.10 (0.14)	0.15 (0.03)
geography PC2	RI	—	0.69	1
	${\hat{\bar{\beta }}}_{\mathrm{st}}$		0.11 (0.09)	0.29 (<0.001)

## 4. Discussion

We studied the regularity of barred plumage in common waxbills with a metric that quantifies the continuity of pigmentation bars within and across feathers [5]. The regularity of barred plumage was positively related to body condition, especially in adult males, indicating that it can convey information on individual quality. Adults had, on average, more regular patterns than juveniles, and adult males had more regular patterns than females, both of which are symptomatic of high regularity having evolved, at least in part, as a sexual signal. We did not find a direct relation between the regularity of barred plumage and colour ornamentation, but some colour traits were related to aspects of individual quality. We discuss these points in turn.

Body condition was positively related to the regularity of barred plumage in adult males. The reported effect size (${\hat{\bar{\beta }}}_{\mathrm{st}}$ on averaged model was 0.15) probably underestimates the information value of barred plumage, because we could only analyse a portion of the birds' barred plumage. In females, the effect size for the relation with body condition was similar to that in males, but our data could not demonstrate a significant relation. Body condition is generally related to the energetic or protein reserves of animals [22] and therefore, within the normal range of variation, gives an indication of individual quality. Similar to our finding, Ferns & Lang [23] and Ferns & Hinsley [24] previously showed that the homogeneity of a colour patch and the straightness of its contour (termed immaculateness by Ferns and co-workers) indicate individual quality in two avian species. The rationale for metrics of immaculateness is similar to that of the metric we used; only the former are tailored to a single and large colour patch, whereas ours is tailored to alternating bars of contrasting colour.

There are at least two distinct ways in which individual quality could be reflected in the regularity of barred plumage. During feather growth some individuals might have been better able to develop regular pigmentation patterns, owing to intrinsic differences in quality or owing to external stressors, whereas others were less resilient to perturbations to feather development. One of the traits we studied, fault bars, is known to reflect stressful conditions during development (reviewed in [25]). The extent of fault bars was not related to the regularity of barred plumage, suggesting that developmental stress is not the main cause for differences in barred plumage among waxbills. Another possibility is that, after feather growth, some individuals have more energy, time and/or skill to maintain plumage in good condition (e.g. with less broken or worn feathers), and therefore are able to maintain a continuous and regular pigmentation pattern. Knowledge on the development and stability of these barred patterns is needed to distinguish among alternative mechanisms linking the regularity of barred plumage to body condition in waxbills.

We did not study if receivers perceive or respond to differences in the regularity of barred plumage. However, indirect evidence suggests they do. The age and sex differences we found (more regular bars in adults than juveniles, and in adult males than females) are identical to those for red colour ornamentation [12,13], and are symptomatic of sexually selected traits in species with conventional sex roles (including mutually ornamented species where adults of both sexes are ornamented, but males more so than females). Adults, and adult males is particular, should not have evolved more elaborate visual traits, whether colour or regularity of bars, unless they are perceivable and affect receiver behaviour. Thus, we suggest that regular barred plumage is, or at least was in the evolutionary past, a communication signal in the common waxbill. In line with a communication function for regular barred patterns, it was shown in other avian species that barred plumage is displayed during social interactions [26], and that receivers respond to experimental manipulations that disrupt the symmetry of barred patterns [27].

Differences in the regularity of barred plumage among individuals were not related to differences in colour ornamentation, and one of the male colour traits was related to a different aspect of quality: the size of male red masks was negatively related to the extent of fault bars. This supports the ‘multiple message hypothesis' for the evolution of multiple ornaments or signals [14]. However, another ornamental trait of males, saturation of red in the bill, was positively related to body condition, similar to the regularity of barred plumage. This supports the ‘redundant message’ hypothesis for the evolution of multiple ornaments or signals: each signal may give a partial indication of condition, and in combination provide more reliable information [14]. Thus, we find some evidence both for distinctiveness and redundancy in the information content of the regularity of barred plumage versus colour ornamentation in waxbills.

The saturation of red in the bill of males was also positively related to feather mite loads. This is unexpected if feather mites are parasitic, in which case infestation should decrease host body condition and, consequently, the expression of ornamentation. A recent meta-analysis indicates that feather mites can be commensal rather than parasitic, and that they can even be more abundant on individuals in better condition [28], which may help explain our finding.

Barred plumage and some colour traits also appeared related to geographical gradients in ecology. Some of these apparent geographical patterns were similar to previously reported changes in ornamentation during this biological invasion by common waxbills (smaller red mask of females in colder, higher-altitude sites [13]), whereas other effects were different (tables 1 and 2). The purpose of including ecological gradients in statistical models was to control for the potentially confounding effect of ecology when testing for relations with the quality of individuals (see Material and methods). As for interpreting apparent ecological effects on barred plumage or colour, these are only suggestive, because effect sizes were small and ecology varies between sites rather than among individuals (and between-site comparisons would yield more conservative results).

In conclusion, we provided the first supporting evidence for the hypothesis that regular barred plumage functions as a sexual signal of quality: the regularity of barred plumage contains information on individual quality, and adult males express more regular pigmentation than females or juveniles, as is typical of sexual signals. Waxbill responses to the regularity of barred plumage have not been studied, but past work on other species has shown that barred plumage can be assessed and actively displayed during social interactions [26,27]. Pigmentation patterns of many kinds can function in camouflage or movement camouflage (e.g. [8,9]; reviewed in [10]), and this appears to be the most widespread function for barred plumage across avian species [6]. We suggest that the regularity of barred plumage also allows using these patterns as communication signals, helping organisms to overcome the functional compromise between camouflage and communication.

## Supplementary Material

Suplementary Tables 1 to 5

## Data accessibility

The dataset supporting this article is available in Dryad at http://dx.doi.org/10.5061/dryad.4j7ss.
