# Supplementary material for: Signalling with a cryptic trait: the regularity of barred plumage in common waxbills
Source: R Soc Open Sci. 2016 May 4;3(5):160195. doi: 10.1098/rsos.160195 (PMC4892462; doi:10.1098/rsos.160195)
Supplement: Suplementary Tables 1 to 5 [file rsos160195supp1.pdf]

SUPPLEMENTARY TABLES TO:
Marques CIJ, Batalha HR & Cardoso GC. Signalling with a cryptic trait: the regularity of barred plumage in common waxbills.

Supplementary Table 1. AICc-based best models (i.e. within 2AICc from the model with lowest AICc) for GLMs relating measures of condition and geographic ecological gradients to the regularity of barred plumage in adult male waxbills. The null model is 6.38 AICc worse than the best model.

Predictors in model	Degrees of freedom	Log-likelihood	AICc	ΔAICc	Akaike weight
Condition + geography PC2	4	-303.57	615.34	0	0.72
Condition + fault bars + geography PC2	5	-303.49	617.27	1.93	0.28

Supplementary Table 2. AICc-based best models for GLMs relating measures of condition and geographic ecological gradients to the regularity of barred plumage in adult female waxbills. The null model is 2.05 AICc worse than the best model.

Predictors in model	Degrees of freedom	Log-likelihood	AICc	ΔAICc	Akaike weight
Condition + geography PC1	4	-257.48	523.19	0	0.22
Condition	3	-258.9	523.94	0.75	0.15
Condition + ecto-parasites + geography PC1	5	-256.84	524.01	0.82	0.14
Condition + fault bars + geography PC1	5	-257.06	524.46	1.26	0.11
Condition + ecto-parasites	4	-258.12	524.47	1.28	0.11
Geography PC1	3	-259.26	524.65	1.46	0.10
Ecto-parasites + geography PC1	4	-258.46	525.14	1.95	0.08
Condition + fault bars	4	-258.47	525.16	1.96	0.08

Supplementary Table 3. AICc-based best models for GLMs relating measures of condition and geographic ecological gradients to bill colour saturation in adult male waxbills. The null model is 9.11 AICc worse than the best model.

Predictors in model	Degrees of freedom	Log-likelihood	AICc	ΔAICc	Akaike weight
Condition + ecto-parasites + geography PC1	5	-122.20	255.11	0	0.70
Condition + fault bars + ecto-parasites + geography PC1	6	-121.91	256.82	1.71	0.30

Supplementary Table 4. AICc-based best models for GLMs relating measures of condition and geographic ecological gradients to the area of the red mask in adult male waxbills. The null model is 6.42 AICc worse than the best model.

Predictors in model	Degrees of freedom	Log-likelihood	AICc	ΔAICc	Akaike weight
Fault bars + geography PC1 + geography PC2	5	-302.51	615.30	0	0.25
Fault bars + geography PC2	4	-303.60	615.39	0.10	0.24
Fault bars + geography PC1	4	-303.96	616.10	0.81	0.17
Fault bars	3	-305.19	616.49	1.19	0.14
Fault bars + ecto-parasite + geography PC1 + geography PC2	6	-302.38	617.15	1.86	0.10
Fault bars + ecto-parasite + geography PC2	5	-303.45	617.18	1.88	0.10

Supplementary Table 5. AICc-based best models for GLMs relating measures of condition and geographic ecological gradients to the area of the red mask in adult female waxbills. The null model is 18.92 AICc worse than the best model.

Predictors in model	Degrees of freedom	Log-likelihood	AICc	ΔAICc	Akaike weight
Fault bars + geography PC1 + geography PC2	5	-247.99	506.31	0	0.35
Condition + Fault bars + geography PC1+ geography PC2	6	-247.19	506.85	0.54	0.27
Geography PC1 + geography PC2	4	-249.40	507.03	0.71	0.24
Condition + geography PC1 + geography PC2	5	-248.89	508.11	1.80	0.14
